# Exact recovery of sparse multiple measurement vectors by $l_{2,p}$-minimization

**DOI:** 10.1186/s13660-017-1601-y

**Published:** 2018-01-10

**Authors:** Changlong Wang, Jigen Peng

**Affiliations:** 1 School of Mathematics and Statistics, Xi’an Jiaotong University, Xi’an, Shaanxi 710049 China; 2 School of Mathematics and Information Science, Guangzhou University, 230 Guangzhou University City Outer Ring Road, Guangzhou, 510006 China

**Keywords:** 15A06, 15A15, 15A48, sparse recovery, multiple measurement vectors, joint sparse recovery, $l_{2,p}$-minimization

## Abstract

The joint sparse recovery problem is a generalization of the single measurement vector problem widely studied in compressed sensing. It aims to recover a set of jointly sparse vectors, i.e., those that have nonzero entries concentrated at a common location. Meanwhile $l_{p}$-minimization subject to matrixes is widely used in a large number of algorithms designed for this problem, i.e., $l_{2,p}$-minimization

$$\begin{aligned} \min_{X \in\mathbb {R}^{n\times r}} \Vert X \Vert _{2,p}\quad \text{s.t. }AX=B. \end{aligned}$$

Therefore the main contribution in this paper is two theoretical results about this technique. The first one is proving that in every multiple system of linear equations there exists a constant $p^{\ast}$ such that the original unique sparse solution also can be recovered from a minimization in $l_{p}$ quasi-norm subject to matrixes whenever $0< p<p^{\ast}$. The other one is showing an analytic expression of such $p^{\ast}$. Finally, we display the results of one example to confirm the validity of our conclusions, and we use some numerical experiments to show that we increase the efficiency of these algorithms designed for $l_{2,p}$-minimization by using our results.

## Introduction

In sparse information processing, one of the central problems is to recover a sparse solution of an underdetermined linear system, such as visual coding [1], matrix completion [2], source localization [3], and face recognition [4]. That is, letting *A* be an underdetermined matrix of size $m \times n$, and $b \in\mathbb{R}^{m}$ is a vector representing some signal, so the single measurement vector (SMV) problem is popularly modeled into the following $l_{0}$-minimization:

1 $$\begin{aligned} \min_{x \in\mathbb {R}^{n}} \Vert x \Vert _{0}\quad\text{s.t. }Ax=b, \end{aligned}$$

where $\Vert x \Vert _{0}$ indicates the number of nonzero elements of *x*.

Furthermore, a natural extension of single measurement vector is the joint sparse recovery problem, also known as the multiple measurement vector (MMV) problem, which arises naturally in source localization [3], neuromagnetic imaging [5], and equalization of sparse-communication channels [6]. Instead of a single measurement *b*, we are given a set of *r* measurements,

2 $$\begin{aligned} Ax^{(k)}=b^{(k)}, \quad k=1, \dots, r, \end{aligned}$$

in which the vectors $x^{(k)}$ ($k=1, \dots, r$) are joint sparse, i.e., the solution vectors share a common support and have nonzero entries concentrated at common locations.

For a given vector *x*, we define a vector function

3 $$\begin{aligned} \Vert x \Vert _{2,0}= \textstyle\begin{cases} 1& \Vert x \Vert _{2}\neq0,\\ 0& \Vert x \Vert _{2}=0. \end{cases}\displaystyle \end{aligned}$$

Then the MMV problem can be modeled as the following $l_{2,0}$-minimization problem:

4 $$\begin{aligned} \min_{X \in\mathbb{R}^{n\times r}} \Vert X \Vert _{2,0}=\sum _{i=1}^{n} \Vert X_{\text{row } i} \Vert _{2,0} \quad\text{s.t. }AX=B, \end{aligned}$$

where $B=[b^{(1)} \dots b^{(r)}] \in\mathbb{R}^{m \times r}$ and $X_{\text{row } i}$ is defined as the *i*th row of *X*.

In this paper, we define the support of *X* by $\operatorname {support}(X)=S=\{i: \Vert X_{\text{row } i} \Vert _{2} \neq 0\}$ and say that the solution *X* is *k*-sparse when $\vert S \vert \leq k$, and we also say that *X* can be recovered by $l_{2,0}$-minimization if *X* is the unique solution of an $l_{2,0}$-minimization problem.

It needs to be emphasized that we cannot regard the solution of multiple measurement vector (MMV) as a combination of several solutions of single measurement vectors, i.e., the solution matrix *X* to $l_{2,0}$-minimization is not always composed of the solution vectors to $l_{0}$-minimization.

### Example 1

We consider an underdetermined system $AX=B$, where

$$A=\left ( \begin{matrix} 2 & 0 & 0 & 1 & 0 \\ 0 & 0.5 & 0 & 1 & 0 \\ 0 & 0 & 1 & 2 & -0.5 \\ 0 & 0 & 0 & -1 & 0.5 \end{matrix} \right )\quad \mbox{and}\quad B=\left ( \begin{matrix} 1 & 1 \\ 1 & 1 \\ 0 & 1 \\ 0 & 0 \end{matrix} \right ). $$

If we treat the $AX=B=[b_{1} \ b_{2} ]$ as a combination of two single measurement vectors $Ax=b_{1}$ and $Ax=b_{2}$, it is easy to verify that each sparse solution to these two problems is $x_{1}=[0.5\ 2\ 0\ 0]^{T}$ and $x_{2}=[0\ 0\ 1\ 2]^{T}$. So let $X^{*}=[x_{1} \ x_{2}]$, it is easy to check that $\Vert X^{*} \Vert _{2,0}=4$. In fact, it is easy to verify that

$$X=\left ( \begin{matrix} 0.5 & 0.5 \\ 2 & 2 \\ 0 & 1 \\ 0 & 0 \end{matrix} \right ) $$

is the solution to $l_{2,0}$-minimization since $\Vert X \Vert _{2,0}=3 < \Vert X^{*} \Vert _{2,0}=4 $.

With this simple Example 1, we should be aware that the MMV problem wants a jointly sparse solution, not a solution which is just composed of sparse vectors. Therefore, the MMV problem is more complex than the SMV one and needs its own theoretical work. Be inspired by $l_{p}$-minimization, a popular approach to find the sparest solution to the MMV problem, which is to solve the following $l_{2,p}$-minimization optimization problem:

5 $$\begin{aligned} \min_{X \in\mathbb {R}^{n\times r}} \Vert X \Vert _{2,p}\quad\text{s.t. }AX=B, \end{aligned}$$

where the mixed norm $\Vert X \Vert _{2,p}^{p}=\sum_{i=1}^{n} \Vert X_{\text{row } i } \Vert _{2}^{p}$.

In [7], $l_{0}$-minimization has been proved to be NP-hard because of the discrete and discontinuous nature of $\Vert x \Vert _{0}$. Therefore, it is obviously NP-hard to solve $l_{2,0}$-minimization too. Due to the fact that $\Vert X \Vert _{2,0}=\lim_{p \to0} \Vert X \Vert _{2,p}^{p}$, it seems to be more natural to consider $l_{2,p}$-minimization instead of an NP-hard optimization $l_{2,0}$-minimization than others.

### Related work

Many researchers have made a lot of contribution related to the existence, uniqueness, and other properties of $l_{2,p}$-minimization (see [8–11]). Eldar [12] gives a sufficient condition for MMV when $p=1$, and Unser [13] analyses some properties of the solution to $l_{2,p}$-minimization when $p=1$. Fourcart and Gribonval [9] studied the MMV setting when $r=2$ and $p=1$; they give a sufficient and necessary condition to judge whether a *k*-sparse matrix *X* can be recovered by $l_{2,p}$-minimization. Furthermore, Lai and Liu [10] consider the MMV setting when $r \geq2$ and $p\in[0,1]$, they improve the condition in [9] and give a sufficient and necessary condition when $r\geq2$.

On the other hand, numerous algorithms have been proposed and studied for $l_{2,0}$-minimization (e.g., [14, 15]). Orthogonal matching pursuit (OMP) algorithms are extended to the MMV problem [16], and convex optimization formulations with mixed norm extend to the corresponding SMV solution [17]. Hyder [15] provides us a robust algorithm for joint sparse recovery, which shows a clear improvement in both noiseless and noisy environments. Furthermore, there exists a lot of excellent work (see[18–22]) presenting us algorithms designed for $l_{2,p}$-minimization. However, it is an important theoretical problem whether there exists a general equivalence relationship between $l_{2,p}$-minimization and $l_{2,0}$-minimization.

In the case $r=1$, Peng [23] has given a definite answer to this theoretical problem. There exists a constant $p(A,b)>0$ such that every solution to $l_{p}$-minimization is also the solution to $l_{0}$-minimization whenever $0< p< p(A,b)$.

However, Peng only proves the conclusion when $r=1$, so it is urgent to extend this conclusion to the MMV problem. Furthermore, Peng just proves the existence of such *p*, he does not give us a computable expression of such *p*. Therefore, the main purpose of this paper is not only to prove the equivalence relationship between $l_{2,p}$-minimization and $l_{2,0}$-minimization, but also present an analysis expression of such *p* in Section 2 and Section 3.

### Main contribution

In this paper, we focus on the equivalence relationship between $l_{2,p}$-minimization and $l_{2,0}$-minimization. Furthermore, it is an application problem that an analytic expression of such $p^{*}$ is needed, especially in designing some algorithms for $l_{2,p}$-minimization.

In brief, this paper gives answers to two problems which urgently need to be solved:

(I). There exists a constant $p^{*}$ such that every *k*-sparse *X* can be recovered by $l_{2,0}$-minimization and $l_{2,p}$-minimization whenever $0< p< p^{*}$.

(II). We give such an analytic expression of such $p^{*}$ based on the restricted isometry property (RIP).

Our paper is organized as follows. In Section 2, we present some preliminaries which play a core role in the proof of our main theorem and prove the equivalence relationship between $l_{2,p}$-minimization and $l_{2,0}$-minimization. In Section 3 we focus on proving another main result of this paper. There we present an analytic expression of such $p^{*}$. Finally, we summarize our findings in the last section.

### Notation

For convenience, for $x \in\mathbb {R}^{n}$, we define its support by $\operatorname{support} (x)=\{i:x_{i} \neq0\}$ and the cardinality of set S by $\vert S \vert $. Let $\operatorname{Ker}(A)=\{x \in\mathbb {R}^{n}:Ax=0\}$ be the null space of matrix A. We also use the subscript notation $x_{S}$ to denote a vector that is equal to *x* on the index set *S* and zero everywhere else and use the subscript notation $X_{S}$ to denote a matrix whose rows are those of the rows of *X* that are in the set index S and zero everywhere else. Let $X_{\text{col } i}$ be the *i*th column in *X*, and let $X_{\text{row } i}$ be the *i*th row in *X*, i.e., $X=[X_{\text{col } 1},X_{\text{col } 2},\dots, X_{\text{col } r}]=[X_{\text{row } 1},X_{\text{row } 2},\dots, X_{\text{row } m}]^{T}$ for $X\in\mathbb{R}^{n \times r}$. We use $\langle A,B\rangle=\operatorname{tr}(A^{T}B)$ and $\Vert A \Vert _{F}=\sum_{i,j} \vert a_{ij} \vert ^{2}$.

## Equivalence relationship between $l_{2,p}$-minimization and $l_{2,0}$-minimization

At the beginning of this section, we introduce a very important property of the measurement matrix *A*.

### Definition 1

([24])

A matrix *A* is said to have the restricted isometry property of order *k* with restricted isometry constant $\delta_{k} \in(0,1)$ if $\delta _{k}$ is the smallest constant such that

6 $$\begin{aligned} (1-\delta_{k}) \Vert x \Vert _{2}^{2} \leq \Vert Ax \Vert _{2}^{2} \leq(1+\delta_{k}) \Vert x \Vert _{2}^{2} \end{aligned}$$

for all *k*-sparse vector *x*, where a vector *x* is said to be *k*-sparse if $\Vert x \Vert _{0}\leq k$.

Next, we will introduce another important concept named M-null space constant, and this concept is the key to proving the equivalence relationship between $l_{2,0}$-minimization and $l_{2,p}$-minimization.

### Definition 2

For $0 \leq p \leq1$ and a positive integer *k*, the M-null space constant $h(p,A,r,k)$ is the smallest number such that

$$\Vert X_{S} \Vert _{2,p}^{p} \leq h(p,A,r,k) \Vert X_{S^{C}} \Vert _{2,p}^{p} $$

for any index set $S \subset\{1,2,\ldots,n\}$ with $\vert S \vert \leq k$ and any $X \in(\operatorname{Ker}(A))^{r}\setminus \{(\textbf{0},\textbf{0},\dots,\textbf{0})\}$.

M-NSC provides us a sufficient and necessary condition of the solution to $l_{2,0}$-minimization and $l_{2,p}$-minimization, and it is important for proving the equivalence relationship between these two models. Furthermore, we emphasize a few important properties of $h(p,A,r,k)$.

### Proposition 1

For a given matrix *A*, the M-NSC $h(p,A,r,k)$ defined in Definition 2 is nondecreasing in $p \in[0,1]$.

### Proof

The proof is divided into two steps.

Step 1: To prove $h(p,A,r,k) \leq h(1,A,r,k)$ for any $p\in[0,1]$.

For any $X \in(N(A))^{r} \setminus \{(\textbf{0},\textbf{0},\dots,\textbf{0})\}$, without loss of generality, we assume that $\Vert X_{\text{row } 1} \Vert _{2} \geq \Vert X_{\text{row } 2} \Vert _{2} \geq\cdots\geq \Vert X_{\text{row } n} \Vert _{2} $.

We define a function $\theta(p,X,k)$ as

7 $$\begin{aligned} \theta(p,X,k)= \frac{\sum_{i=1}^{k} \Vert X_{\text{row } i} \Vert _{2}^{p}}{\sum_{i=k+1}^{n} \Vert X_{\text{row } i} \Vert _{2}^{p}}, \end{aligned}$$

then it is easy to get that the definition of $h(p,A,r,k)$ is equivalent to

8 $$\begin{aligned} h(p,A,r,k)=\max_{ \vert S \vert \leq k} \sup_{X \in (N(A))^{r} \setminus \{(\textbf{0},\textbf{0}, \dots,\textbf{0} )\}} \theta(p,X,k). \end{aligned}$$

For any $p \in[0,1]$, we notice that the function $f(t)=\frac {t^{p}}{t}$ ($t>0$) is a nonincreasing function. For any $j \in\{ k+1,\dots, n \}$ and $i \in\{1,2,\dots, k\}$, we have that

9 $$\begin{aligned} \frac{ \Vert X_{\text{row } j} \Vert _{2}^{p}}{ \Vert X_{\text{row } j} \Vert _{2}} \geq\frac{ \Vert X_{\text{row } i} \Vert _{2}^{p}}{ \Vert X_{\text{row } i} \Vert _{2}}. \end{aligned}$$

We can rewrite inequalities (9) into

10 $$\begin{aligned} \frac{ \Vert X_{\text{row } i} \Vert _{2}^{p}}{ \Vert X_{\text{row } j} \Vert _{2}^{p}} \leq\frac{ \Vert X_{\text{row } i} \Vert _{2}}{ \Vert X_{\text{row } j} \Vert _{2}}. \end{aligned}$$

Therefore, we can get that

11 $$\begin{aligned} \frac{\sum_{i=1}^{k} \Vert X_{\text{row } i} \Vert _{2}^{p}}{ \Vert X_{\text{row } j} \Vert _{2}^{p}} \leq\frac{\sum_{i=1}^{k} \Vert X_{\text{row } i} \Vert _{2}}{ \Vert X_{\text{row } j} \Vert _{2}}. \end{aligned}$$

We can conclude that

12 $$\begin{aligned} \frac{\sum_{j=k+1}^{n} \Vert X_{\text{row } j} \Vert _{2}^{p}}{\sum_{i=1}^{k} \Vert X_{\text{row } i} \Vert _{2}^{p}} \geq\frac{\sum_{j=k+1}^{n} \Vert X_{\text{row } j} \Vert _{2}}{\sum_{i=1}^{k} \Vert X_{\text{row } i} \Vert _{2}} \end{aligned}$$

such that $\frac{1}{\theta(p,X,k)} \geq\frac{1}{\theta(1,X,k)}$, i.e., $\theta(p,X,k) \leq\theta(1,X,k)$.

Because $h(p,A,r,k)=\max_{ \vert S \vert \leq k} \sup_{X \in(N(A))^{r} \setminus \{(\textbf{0},\textbf{0}, \dots)\}} \theta (p,X,k)$, we can get that $h(p,A,r,k) \leq h(1,A,r,k)$.

Step 2: To prove $h(pq,A,r,k) \leq h(p,A,r,k)$ for any $p\in[0,1]$ and $q\in(0,1)$.

According to the definition of $\theta(p,X,k)$ in Step 1, we have that

13 $$\begin{aligned} \theta(pq,X,k)=\frac{\sum_{i=1}^{k} \Vert X_{\text{row } i} \Vert _{2}^{pq}}{\sum_{j=k+1}^{n} \Vert X_{\text{row } j} \Vert _{2}^{pq}}=\frac{\sum_{i=1}^{k}( \Vert X_{\text{row } i} \Vert _{2}^{p})^{q}}{\sum_{j=k+1}^{n}( \Vert X_{\text{row } j} \Vert _{2}^{p})^{q}} \leq\frac{\sum_{i=1}^{n} \Vert X_{\text{row } i} \Vert _{2}^{p}}{\sum_{j=k+1}^{n} \Vert X_{\text{row } j} \Vert _{2}^{p}}. \end{aligned}$$

It needs to be pointed out that we have proved the fact in Step 1 that

14 $$\begin{aligned} \frac{\sum_{i=1}^{k} \vert u_{i} \vert ^{p}}{\sum_{j=k+1}^{n} \vert u_{j} \vert ^{p}} \leq\frac{\sum_{i=1}^{k} \vert u_{i} \vert }{\sum_{j=k+1}^{n} \vert u_{j} \vert } \end{aligned}$$

for any $\vert u_{1} \vert \geq \vert u_{2} \vert \dots \vert u_{n} \vert $.

Therefore, we can get that $\theta(pq,X,k)\leq\theta(p,X,k)$; in other words, $\theta(p_{1},X,k)\leq\theta(p_{2},X,k)$ as long as $p_{1} \leq p_{2}$.

Because $h(p,A,r,k)=\max_{ \vert S \vert \leq k} \sup_{X \in(N(A))^{r} \setminus \{0,0, \dots,0\}} \theta(p,X,k)$, so we can get that $h(p,A,r,k)$ is nondecreasing in $p \in[0,1]$

The proof is completed. □

### Proposition 2

For a given matrix *A*, the M-NSC $h(p,A,r,k)$ defined in Definition 2 is a continuous function in $p \in[0,1]$

### Proof

As has been proved in Proposition 1, $h(p,A,r,k)$ is nondecreasing in $p \in[0,1]$ such that there is jump discontinuous if $h(p,A,r,k)$ is discontinuous at a point. Therefore, it is enough to prove that it is impossible to have jump discontinuous points of $h(p,A,r,k)$.

For convenience, we still use $\theta(p,X,S)$ which is defined in the proof of Proposition 1, and the following proof is divided into three steps.

Step 1. To prove that there exist $X\in(N(A))^{r}$ and a set $S \subset \{1,2,\dots, n\}$ such that $\theta(p,X,S)=h(p,A,r,k)$.

Let $V=\{X\in((N(A))^{r}): \Vert X_{\text{row } i} \Vert _{2}=1, i=1,2,\dots, n\}$, and it is easy to get that $h(p,A,r,k)=\max_{ \vert S \vert \leq k} \sup_{X \in V} \theta(p,X,S)$

It needs to be pointed out that the choice of the set $S\subset {1,2,\dots, n}$ with $\vert S \vert \leq k$ is limited, so there exists a set $S^{\prime}$ with $\vert S^{\prime} \vert \leq k$ such that $h(p,A,r,k)=\sup_{X \in V} \theta(p,X,S^{\prime})$.

On the other hand, $\theta(p,X,S^{\prime})$ is obviously continuous in *X* on *V*. Because of the compactness of *V*, there exists $X^{\prime}\in V$ such that $h(p,A,r,k)=\theta(p,X^{\prime},S^{\prime})$.

Step 2. To prove that $\lim_{p \to p_{0}^{-}} h(p,A,r,k)=h(p_{0},A,r,k)$.

We assume that $\lim_{p \to p_{0}^{-}} h(p,A,r,k) \ne h(p_{0},A,r,k)$. According to Proposition 1, $h(p,A, r,k)$ is nondecreasing in $p \in[0,1]$, therefore, we can get a sequence of $\{p_{n}\}$ with $p_{n} \to p_{0}^{-}$ such that

15 $$\begin{aligned} \lim_{p_{n} \to p_{0}^{-}} h(p_{n},A,r,k)=M< h(p_{0},a,r,k). \end{aligned}$$

According to the proof in Step 1, there exist $X^{\prime}\in(N(A))^{r}$ and $S \subset\{1,2,\dots, n\}$ such that $h(p_{0},A,r,k)=\theta (p_{0},X^{\prime},S^{\prime})$. It is easy to get that $\lim_{p \to p_{0}^{-}} \theta(p_{n},X,S^{\prime})=\theta(p,X^{\prime},S^{\prime})=h(p_{0},A,r,k)$.

However, according to the definition of $\theta(p,X,S)$, it is obvious that

16 $$\begin{aligned} h(p_{n},A,r,k) \geq\theta\bigl(p_{n},X^{\prime},S^{\prime} \bigr); \end{aligned}$$

however, (15) and (16) contradict each other.

Therefore, we have that $\lim_{p \to p_{0}^{-}} h(p,A,r,k)=h(p_{0},A,r,k)$.

Step 3. To prove that $\lim_{p \to p_{0}^{+}} h(p,A,r,k)=h(p_{0},A,r,k)$ for any $p_{0}\in[0,1)$.

We consider a sequence of $\{p_{n}\}$ with $p_{0} \leq p_{n}<1$ and $p \to p_{0}^{+}$.

According to Step 1, there exist $X_{n} \in V$ and $\vert S_{n} \vert \leq k$ such that $h(p_{n},A,r,k)=\theta(p_{n},X_{n},S_{n})$. Since the choice of $S\subset\{1,2,\dots, n\}$ with $\vert S \vert \leq k$ is limited, there exist two subsequences $\{p_{n_{i}}\}$ of $\{ p_{n}\}$, $\{X_{n_{i}}\}$ of $\{X_{n}\}$ and a set $S^{\prime}$ such that $\theta (p_{n_{i}},X_{n_{i}},S^{\prime})=h(p_{n_{i}},A,r,k)$.

Furthermore, since $X_{n} \in V$, it is easy to get a subsequence of $X_{n_{i}}$ which is convergent. Without loss of generality, we assume that $X_{n_{i}} \to X^{\prime}$.

Therefore, we can get that $h(p_{n_{i}},A,r,k)=\theta (p_{n_{i}},X_{n_{i}},S^{\prime}) \to \theta(p_{0},X^{\prime},S^{\prime})$.

According to the definition of $h(p_{0},A,r,k)$, we can get that $\theta (p_{0},X^{\prime},S^{\prime}) \leq h(p_{0},A,r,k)$ such that $\lim_{p \to p_{0}^{+}} h(p,A,r,k)=h(p_{0},A,r,k)$.

Combining Step 2 and Step 3, we show that it is impossible for $h(p,A,r,k)$ to have jump discontinuous.

The proof is completed. □

**Figure 1:**
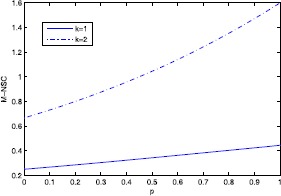
**M-NSC in Example** **1** **.**

The concept M-NSC is very important in this paper and it will offer tremendous help in illustrating the performance of $l_{2,0}$-minimization and $l_{2,p}$-minimization; however, M-NSC is difficult to be calculated for a large scale matrix. We show the figure of M-NSC in Example 1 in Figure 1. Combining Propositions 1 and 2, we can get the first main theorem which shows us the equivalence relationship between $l_{2,0}$-minimization and $l_{2,p}$-minimization.

### Theorem 1

*If every* *k*-*sparse matrix* *X* *can be recovered by* $l_{2,0}$-*minimization*, *then there exists a constant* $p^{*}(A,B,r)$ *such that* *X* *also can be recovered by* $l_{2,p}$-*minimization whenever* $0< p< p^{*}(A,B,r)$.

### Proof

First of all, we will prove that $h(0,A,r,k)<1$ under the assumption. If $h(0,A,r,k)\geq1$ for some fixed *r* and *k*, then there exists $X\in(N(A))^{r}$ such that $\Vert X_{S} \Vert _{2,0}\geq \Vert X_{S^{C}} \Vert _{2,0}$ for a certain set $S\subset\{ 1,2,\ldots,n\}$ with $\vert S \vert \leq k$. Let $B=AX_{S}$, then it is obvious that $X_{S^{C}}$ is a sparser solution than $X_{S}$, which contradicts the assumption.

By Propositions 1 and 2, since $h(p,A,r,k)$ is continuous and nondecreasing at the point $p=0$, there exist a constant $p^{*}(A,B,r)$ and a small enough number *δ* that $h(0,A,r,k)< h(p,A,r,k)\leq h(0,A,r,k)+\delta< 1$ for any $p \in (0,p^{*}(A,B,r))$.

Therefore, for a given *k*-sparse matrix $X^{*}\in\mathbb {R}^{n\times r}$ and any $0< p< p^{*}(A,B,r)$, we have that

17 $$\begin{aligned} \bigl\Vert X^{*}+H \bigr\Vert _{2,p}^{p} =& \bigl\Vert \bigl(X^{*}+H\bigr)_{S^{*}} \bigr\Vert _{2,p}^{p}+ \Vert H_{S^{*C}} \Vert _{2,p}^{p} \\ \geq& \bigl\Vert X^{*} \bigr\Vert _{2,p}^{p}- \Vert H_{S^{*}} \Vert _{2,p}^{p}+ \Vert H_{S^{*C}} \Vert _{2,p}^{p}> \bigl\Vert X^{*} \bigr\Vert _{2,p}^{p}. \end{aligned}$$

The last inequality is the result from the inequality $h(p,A,r,k)<1$, the proof is completed. □

## An analysis expression of such p

In Section 2, we have proved the fact that there exists a constant $p^{*}(A,B,r)$ such that both $l_{2,p}$-minimization and $l_{2,0}$-minimization have the same solution. However, it is also important to give such an analytic expression of $p^{*}(A,B,r)$. In Section 3, we focus on giving an analytic expression of an upper bound of $h(p,A,r,k)$, and we can get the equivalence relationship between $l_{2,p}$-minimization and $l_{2,0}$-minimization as long as $h(p,A,r,k)<1$ is satisfied. In order to reach our goal, we postpone our main theorems and begin with some lemmas.

### Lemma 1

*For any* $X\in\mathbb{R}^{n \times r}$ *and* $p\in(0,1]$, *we have that*

$$\Vert X \Vert _{2,p} \leq \Vert X \Vert _{2,0}^{\frac{1}{p}-\frac{1}{2}} \Vert X \Vert _{F}. $$

### Proof

For any $X \in\mathbb{R}^{n \times r}$, without loss of generality, we assume that $\Vert X_{\text{row } i} \Vert _{2} =0$ for $i \in\{ \Vert X \Vert _{2,0}+1,\ldots,n\}$.

According to Hölder’s inequality, we can show that

18 $$\begin{aligned} \Vert X \Vert _{2,p}^{p}=\sum _{i=1}^{ \Vert X \Vert _{2,0}} \Vert X_{\text{row } i} \Vert _{2}^{p} \leq \Biggl(\sum_{i=1}^{ \Vert X \Vert _{2,0}} \bigl( \Vert X_{\text{row } i} \Vert _{2}^{p} \bigr)^{\frac{2}{p}} \Biggr)^{\frac {p}{2}} \Biggl(\sum _{i=1}^{ \Vert X \Vert _{2,0}} 1 \Biggr)^{1-\frac{p}{2}}= \Vert X \Vert _{2,0}^{1-\frac {p}{2}} \Vert X \Vert _{F}^{p}, \end{aligned}$$

that is, $\Vert X \Vert _{2,p} \leq \Vert X \Vert _{2,0}^{\frac{1}{p}-\frac{1}{2}} \Vert X \Vert _{F}$. □

### Lemma 2

([25])

*If* $0< p< q$ *and* $u_{1}\geq\cdots\geq u_{k}\geq u_{k+1}\geq\cdots\geq u_{s} \geq u_{s+1}\geq\cdots\geq u_{k+t}\geq0$, *it holds that*

19 $$\begin{aligned} \Biggl(\sum_{i=k+1}^{k+t}u_{i}^{q} \Biggr)^{\frac {1}{q}}\leq C_{p,q}(k,s,t) \Biggl(\sum _{i=1}^{s} u_{i}^{p} \Biggr)^{\frac{1}{p}} \end{aligned}$$

*with* $C_{p,q}(k,s,t)=\max \{\frac{t^{\frac {p}{q}}}{s}, (\frac{p}{q} )^{\frac{p}{q}} (1-\frac {p}{q} )^{1-\frac{p}{q}}k^{\frac{p}{q}-1} \}^{\frac{1}{p}}$.

### Lemma 3

*For* $p\in(0,1]$, *we have that* $(\frac{p}{2} )^{\frac{1}{2}} (\frac{1}{2-p} )^{\frac{1}{2}-\frac {1}{p}}\geq\frac{\sqrt{2}}{2}$.

### Proof

We denote a function $f(p)$ on the interval $(0,1]$

20 $$\begin{aligned} f(p)= \biggl(\frac{p}{2} \biggr)^{\frac{1}{2}} \biggl(\frac{1}{2-p} \biggr)^{\frac{1}{2}-\frac{1}{p}}, \end{aligned}$$

and we can get that

21 $$\begin{aligned} \ln f(p)=\frac{1}{2}\ln\frac{p}{2}- \biggl(\frac {1}{2}- \frac{1}{p} \biggr)\ln(2-p). \end{aligned}$$

It is easy to get that

22 $$\begin{aligned} h(p) =&\frac{f'(p)}{f(p)}=\frac{1}{2p}- \biggl(\frac {1}{p^{2}} \ln(2-p)- \biggl(\frac{1}{2}-\frac{1}{p} \biggr)\frac {1}{2-p} \biggr) \\ =&-\frac{1}{p^{2}}\ln(2-p)\leq0. \end{aligned}$$

Therefore, $f(p)$ is nondecreasing in $p \in(0,1]$, and we can get $f(p)\geq f(1)=\frac{\sqrt{2}}{2}$.

The proof is completed. □

### Lemma 4

*For any* $0< p\leq1$, *we have that* $(1-\frac{p}{2} )^{\frac {1}{p}-\frac{1}{2}}\leq (\frac{\sqrt{2}}{2}-e^{-\frac {1}{2}} )p+e^{-\frac{1}{2}}$.

### Proof

We define a function $\varphi(p)$ on the interval $(0,1]$ by $\varphi (p)= (1-\frac{p}{2} )^{\frac{1}{p}-\frac{1}{2}}$, and it is easy to get that

23 $$\begin{aligned} \ln\varphi(p)= \biggl(\frac{1}{p}-\frac{1}{2} \biggr)\ln \biggl(1-\frac{p}{2} \biggr). \end{aligned}$$

Now, we take the derivative of both sides of inequality (23) with respect to *p*, it is easy to get that

24 $$\begin{aligned} \frac{\varphi'(p)}{\varphi(p)} =&-\frac{1}{p^{2}}\ln \biggl(1-\frac{p}{2} \biggr)+ \biggl(\frac{1}{p}-\frac{1}{2} \biggr)\frac {-1}{2-p} \\ =&\frac{1}{p} \biggl(\frac{1}{p}\ln\frac{2}{2-p}- \frac {1}{2} \biggr) \\ =&\frac{1}{p} \biggl(\frac{1}{p}\ln \biggl(1+ \frac {p}{2-p} \biggr)-\frac{1}{2} \biggr) \\ \geq&\frac{1}{p} \biggl(\frac{1}{p} \biggl( \frac {p}{2-p}+\frac{p^{2}}{2(2-p)^{2}} \biggr)-\frac{1}{2} \biggr) \\ =& \frac{1}{p} \biggl(\frac{4-p}{2(2-p)^{2}}-\frac{1}{2} \biggr)>0. \end{aligned}$$

The fourth inequality is due to the fact that $\ln(1+x)\leq x-\frac {x^{2}}{2}$ for any $x\in(0,1]$. The last inequality is the result from a simple inequality $4-p-(2-p)^{2}=3p-p^{2}>0$.

Because $\lim_{p\rightarrow0} \varphi(p)=e^{-\frac{1}{2}}$ and $\varphi(1)=\frac{\sqrt{2}}{2}$, we can get that $\varphi(p)$ is an increasing function and $\varphi(p)\leq\frac{\sqrt{2}}{2}$ for $0< p\leq1$.

In fact, we have got the result $\varphi^{\prime}(p)= (\frac {1}{p^{2}}\ln (1+\frac{p}{2-p} )-\frac{1}{2p} )\cdot \varphi(p)$, and it is easy to get

25 $$\begin{aligned} \varphi^{\prime\prime}(p) =& \biggl( \biggl(\frac{-2}{p}+ \frac {1}{p^{2}} \biggr) \bigl(\ln2-\ln(2-p)\bigr)+\frac{1}{p^{2}(2-p)}+ \frac {1}{2p^{2}}-\frac{1}{2p} \biggr)\cdot\varphi(p) \\ =& \frac{1}{p}\cdot g(p) \cdot\varphi(p), \end{aligned}$$

where $g(p)=\frac{1}{p(2-p)}+\frac{1}{2p}- (\frac{2-p}{p^{2}}\ln (1+\frac{p}{2-p} )+\frac{1}{2} )$.

Due to the fact $\ln(1+x)\leq x-\frac{x^{2}}{2}+\frac{x^{3}}{3}$, we have that

26 $$\begin{aligned} \ln \biggl(1+\frac{p}{2-p} \biggr)+\frac{1}{2} \leq& \frac {p}{2-p}-\frac{p^{2}}{2(2-p)}+\frac{p^{3}}{3(2-p)}+\frac{1}{2}, \\ =& \frac{3p^{3}-p^{2}-p+24}{6p(2-p)^{2}}. \end{aligned}$$

Furthermore, it is easy to get that $\frac{1}{p(2-p)}+\frac {1}{2p}=\frac{24-18p+3p^{2}}{6p(2-p)^{2}}$ and $g(p)\geq0$ since $4>3p$.

Therefore, $\varphi(p)$ is an increasing convex function in $(0,1]$. Furthermore, it is easy to get that $\varphi(0)=e^{-\frac{1}{2}}$ and $\varphi(1)=\frac{\sqrt{2}}{2}$. By the property of the convex function, we can conclude that

27 $$\begin{aligned} \biggl(1-\frac{p}{2} \biggr)^{\frac{1}{p}-\frac{1}{2}}\leq \biggl( \frac{\sqrt{2}}{2}-e^{-\frac{1}{2}} \biggr)p+e^{-\frac{1}{2}}. \end{aligned}$$

 □

### Lemma 5

*Given an underdetermined matrix* $A \in\mathbb{R}^{m \times n}$ *which satisfies RIP of order* 2*k*, *we have that*

28 $$\begin{aligned} \bigl\vert \langle AX_{1},AX_{2}\rangle \bigr\vert \leq \delta_{2k} \Vert X_{1} \Vert _{F} \Vert X_{2} \Vert _{F} \end{aligned}$$

*for any* *k*-*sparse matrices* $X_{1},X_{2} \in\mathbb{R}^{n \times r}$ *with* $\operatorname{support}(X_{1}) \cap\operatorname{support}(X_{2})= \varnothing$.

### Proof

According to the definition of inner product of matrixes, it is easy to get that

29 $$\begin{aligned} \bigl\Vert A(X_{1}+X_{2}) \bigr\Vert _{F}^{2}=\bigl\langle A(X_{1}+X_{2}),A(X_{1}+X_{2}) \bigr\rangle \end{aligned}$$

and

30 $$\begin{aligned} \biggl\Vert \frac{1}{\Vert X_{1}\Vert_{F}}X_{1}+\frac{1}{\Vert X_{2}\Vert _{F}}X_{2} \biggr\Vert _{F}^{2} = \biggl\Vert \frac{1}{\Vert X_{1}\Vert_{F}}X_{1}- \frac{1}{\Vert X_{2}\Vert _{F}}X_{2} \biggr\Vert _{F}^{2}=2 \end{aligned}$$

since $\operatorname{support}(X_{1}) \cap\operatorname{support}(X_{2})= \varnothing$.

Therefore, we can get that

31 $$\begin{aligned} \frac{ \vert \langle AX_{1},AX_{2} \rangle \vert }{ \Vert X_{1} \Vert _{F} \Vert X_{2} \Vert _{F}} =& \biggl\vert \biggl\langle A\frac{1}{ \Vert X_{1} \Vert _{F}}X_{1},A \frac{1}{ \Vert X_{2} \Vert _{F}}X_{2} \biggr\rangle \biggr\vert \\ =&\frac{1}{4} \biggl\vert \biggl\Vert A\frac{1}{\Vert X_{1}\Vert _{F}}X_{1}+A \frac{1}{\Vert X_{2}\Vert_{F}}X_{2} \biggr\Vert _{F}^{2}- \biggl\Vert A\frac{1}{\Vert X_{1}\Vert_{F}}X_{1}-A\frac{1}{\Vert X_{2}\Vert_{F}}X_{2} \biggr\Vert _{F}^{2} \biggr\vert \\ \leq& \frac{1}{4} \bigl((1+\delta_{2k})\cdot2-(1-\delta _{2k})\cdot2 \bigr) \\ =& \delta_{2k}. \end{aligned}$$

The proof is completed. □

Now, we present another main contribution in this paper.

### Theorem 2

*Given an underdetermined matrix* $A\in\mathbb{R}^{m\times n}$ *which satisfies RIP of order* 2*k*, *for any* $p\in(0,1]$, *we can get an upper bound of* $h(p,A,r,k)$.

32 $$\begin{aligned} h(p,A,r,k)\leq M(\delta_{2k},p)= \biggl(\frac{(\sqrt{2}+1)\delta _{2k}}{1-\delta_{2k}} \biggl( \frac{p}{2} \biggr)^{\frac{1}{2}} \biggl(1-\frac{p}{2} \biggr)^{\frac{1}{p}-\frac{1}{2}} \biggr)^{p}. \end{aligned}$$

### Proof

For any $X \in(N(A))^{r} \setminus\{(\textbf{0},\textbf{0},\dots, \textbf{0})\}$, we define a vector $x\in\mathbb {R}^{n}$ as

33 $$\begin{aligned} x= \bigl[ \Vert X_{\text{row } 1} \Vert _{2}, \Vert X_{\text{row } 2} \Vert _{2},\dots, \Vert X_{\text{row } n} \Vert _{2}\bigr]^{T}, \end{aligned}$$

and we consider the index set

$$\begin{aligned}& S_{0}=\{ \mbox{indices of the largest }k\mbox{ values component of }x \}, \\& S_{1}=\{ \mbox{indices of the largest }k1\mbox{ values component of }x\mbox{ except }S_{0}\}, \\& S_{2}=\{ \mbox{indices of the largest }k\mbox{ values component of }x \mbox{ except }S_{0}\mbox{ and }S_{1}\}, \\& \dots \\& S_{t}=\{ \mbox{indices of the rest components of }x \}. \end{aligned}$$

Since $X \in(N(A))^{r}$, it is obvious that

34 $$\begin{aligned} AX=A(X_{S_{0}}+X_{S_{1}}+\cdots+X_{S_{t}})=(\textbf{0}, \textbf{0},\dots, \textbf{0}), \end{aligned}$$

and it is easy to get that

35 $$\begin{aligned} \bigl\Vert A(X_{S_{0}}+X_{S_{1}}) \bigr\Vert _{F}^{2} =&\bigl\langle A(X_{S_{0}}+X_{S_{1}}), -A(X_{S_{2}}+X_{S_{3}}+\cdots+X_{S_{t}})\bigr\rangle \\ \leq&\delta_{2k}\bigl( \Vert X_{S_{0}} \Vert _{F}+ \Vert X_{S_{1}} \Vert _{F}\bigr)\cdot\sum _{i=2}^{t} \Vert X_{S_{i}} \Vert _{F}. \end{aligned}$$

The second inequality uses the result from Lemma 5. Next, we will give an estimate of $\sum_{i=2}^{t} \Vert X_{S_{i}} \Vert _{F}$. On the one hand, by (35), it is obvious that

36 $$\begin{aligned} \Vert X_{S_{0}} \Vert _{F}^{2}+ \Vert X_{S_{1}} \Vert _{F}^{2} =& \Vert X_{S_{0}}+X_{S_{1}} \Vert _{F}^{2} \\ \leq& \frac{1}{1-\delta_{2k}} \bigl\Vert A(X_{S_{0}}+X_{S_{1}}) \bigr\Vert _{F}^{2} \\ \leq& \frac{\delta_{2k}}{1-\delta_{2k}}\bigl( \Vert X_{S_{0}} \Vert _{F}+ \Vert X_{S_{1}} \Vert _{F}\bigr)\cdot\sum _{i=2}^{t} \Vert X_{S_{i}} \Vert _{F}. \end{aligned}$$

On the other hand, we have that

37 $$\begin{aligned} \Vert X_{S_{i}} \Vert _{F}+ \Vert X_{S_{i+1}} \Vert _{F}+ \Vert X_{S_{i+2}} \Vert _{F}+ \Vert X_{S_{i+3}} \Vert _{F}\leq2\cdot \Vert X_{S_{i}\cup S_{i+1}\cup S_{i+2}\cup S_{i+3}} \Vert _{F} \end{aligned}$$

for any $2\leq i \leq t$. Therefore,

38 $$\begin{aligned} \sum_{i=2}^{t} \Vert X_{S_{i}} \Vert _{F} \leq2\cdot\sum_{i\geq0} \Vert X_{S_{4i+2}\cup S_{4i+3}\cup S_{4i+4}\cup S_{4i+5}} \Vert _{F}. \end{aligned}$$

By Lemma 2, we can get that

39 $$\begin{aligned} \Vert X_{S_{i}\cup S_{i+1}\cup S_{i+2} \cup S_{i+3}} \Vert _{F}\leq C(p) \Vert X_{S_{i-1}\cup S_{i}\cup S_{i+1} \cup S_{i+2}} \Vert _{2,p}, \end{aligned}$$

where

$$\begin{aligned} C(p) =&\max\biggl\{ (4k)^{\frac{1}{2}-\frac{1}{p}}, \biggl(\frac {p}{2} \biggr)^{\frac{1}{2}} (2-p )^{\frac{1}{p}-\frac {1}{2}}(2k)^{\frac{1}{2}-\frac{1}{p}}\biggr\} . \end{aligned}$$

By Lemma 3, we can conclude that

40 $$\begin{aligned} C(p)= \biggl(\frac{p}{2} \biggr)^{\frac{1}{2}} (2-p )^{\frac{1}{p}-\frac{1}{2}}(2k)^{\frac{1}{2}-\frac{1}{p}} \end{aligned}$$

since $(\frac{p}{2} )^{\frac{1}{2}} (\frac {1}{2-p} )^{\frac{1}{2}-\frac{1}{p}}\geq\frac{\sqrt{2}}{2}$.

Therefore, we can get that

41 $$\begin{aligned} \sum_{i=2}^{t} \Vert X_{S_{i}} \Vert _{F} \leq& 2 \sum _{i\geq0} \Vert X_{S_{4i+2}\cup S_{4i+3}\cup S_{4i+4} \cup S_{4i+5}} \Vert _{F} \\ \leq& 2 \cdot \biggl(\frac{p}{2} \biggr)^{\frac{1}{2}} (2-p )^{\frac{1}{p}-\frac{1}{2}}(2k)^{\frac{1}{2}-\frac {1}{p}}\sum_{i\geq0} \Vert X_{S_{4i+1}\cup S_{4i+2}\cup S_{4i+3} \cup S_{4i+4}} \Vert _{2,p} \\ \leq& 2 \cdot \biggl(\frac{p}{2} \biggr)^{\frac{1}{2}} (2-p )^{\frac{1}{p}-\frac{1}{2}}(2k)^{\frac{1}{2}-\frac {1}{p}} \Vert X_{S_{0}^{C}} \Vert _{2,p}. \end{aligned}$$

Therefore, we have that

42 $$\begin{aligned} \Vert X_{S_{0}} \Vert _{F}^{2}+ \Vert X_{S_{1}} \Vert _{F}^{2} \leq& \frac{\delta_{2k}}{1-\delta_{2k}}\bigl( \Vert X_{S_{0}} \Vert _{F}+ \Vert X_{S_{1}} \Vert _{F}\bigr) \Biggl(\sum _{i=2}^{t} \Vert X_{S_{i}} \Vert _{F} \Biggr) \\ \leq& \Phi\cdot\bigl( \Vert X_{S_{0}} \Vert _{F}+ \Vert X_{S_{1}} \Vert _{F}\bigr), \end{aligned}$$

where $\Phi=\frac{2\delta_{2k}}{1-\delta_{2k}} (\frac {p}{2} )^{\frac{1}{2}} (2-p )^{\frac{1}{p}-\frac {1}{2}}(2k)^{\frac{1}{2}-\frac{1}{p}} \Vert X_{S_{0}^{C}} \Vert _{2,p}$.

By (42), it is easy to get that

43 $$\begin{aligned} \biggl( \Vert X_{S_{0}} \Vert _{F}-\frac{\Phi}{2} \biggr)^{2}+ \biggl( \Vert X_{S_{1}} \Vert _{F}- \frac{\Phi}{2} \biggr)^{2}\leq\frac{\Phi^{2}}{2}. \end{aligned}$$

Therefore, we can get that

44 $$\begin{aligned} \Vert X_{S_{0}} \Vert _{F}\leq\frac{\sqrt{2}+1}{2} \Phi. \end{aligned}$$

By Lemma 1,

45 $$\begin{aligned} \Vert X_{S_{0}} \Vert _{2,p}\leq k^{\frac{1}{p}-\frac {1}{2}} \Vert X_{S_{0}} \Vert _{F}\leq k^{\frac{1}{p}-\frac {1}{2}} \frac{\sqrt{2}+1}{2} \Phi. \end{aligned}$$

By the definition of M-NSC, we can conclude that

46 $$\begin{aligned} h(p,A,r,k)\leq M(\delta_{2k},p)= \biggl(\frac{(\sqrt{2}+1)\delta _{2k}}{1-\delta_{2k}} \biggl( \frac{p}{2} \biggr)^{\frac{1}{2}} \biggl(1-\frac{p}{2} \biggr)^{\frac{1}{p}-\frac{1}{2}} \biggr)^{p}. \end{aligned}$$

 □

### Theorem 3

*Given a matrix* $A\in R^{m\times n}$ *with* $m \leq n$, *every* *k*-*sparse* $X^{\ast}$ *can also be recovered by* $l_{2,p}$-*minimization*, *for any* $0< p\leq p^{\ast}(A)$, *where*

47 $$\begin{aligned} p^{*}(A)= \biggl(\sqrt[3]{-\frac{w_{2}}{2}+\sqrt{\frac{w_{2}^{2}}{4}+ \frac {w_{1}^{3}}{27}}}+\sqrt[3]{-\frac{w_{2}}{2}-\sqrt{ \frac{w_{2}^{2}}{4}+\frac {w_{1}^{3}}{27}}} \biggr)^{2}, \end{aligned}$$

*and* $w_{1}=\frac{e^{-\frac{1}{2}}}{\frac{\sqrt{2}}{2}-e^{-\frac {1}{2}}}$ *and* $w_{2}=-\frac{(1-\delta_{2k})^{2}}{(2+\sqrt{2})(\frac {\sqrt{2}}{2}-e^{-\frac{1}{2}})\delta_{2k}}$.

### Proof

According to Theorem 2, we can get the equivalence between $l_{2,0}$-minimization and $l_{2,p}$-minimization as soon as $M(\delta _{2k},p)<1$, where $M(\delta_{2k},p)$ is defined in Theorem 2.

48 $$\begin{aligned} \frac{(\sqrt{2}+1)\delta_{2k}}{1-\delta_{2k}} \biggl(\frac {p}{2} \biggr)^{\frac{1}{2}} \biggl(1- \frac{p}{2} \biggr)^{\frac {1}{p}-\frac{1}{2}}< 1. \end{aligned}$$

However, we realize the inequality $M(\delta_{2k},p)<1$ is difficult to solve, so we need to change this equality into another form. By Lemma 4, it is easy to get that

49 $$\begin{aligned} M(\delta_{2k},p)\leq\frac{(\sqrt{2}+2)\delta_{2k}}{2(1-\delta _{2k})}\sqrt{p} \biggl( \biggl( \frac{\sqrt{2}}{2}-e^{-\frac {1}{2}} \biggr)p+e^{-\frac{1}{2}} \biggr). \end{aligned}$$

Let $\sqrt{p}=\eta$, and we consider the following inequality:

50 $$\begin{aligned} \eta^{3}+w_{1}\eta+w_{2}< 0, \end{aligned}$$

where $w_{1}=\frac{e^{-\frac{1}{2}}}{\frac{\sqrt{2}}{2}-e^{-\frac {1}{2}}}$ and $w_{2}=-\frac{(1-\delta_{2k})^{2}}{(2+\sqrt{2})\delta _{2k}(\frac{\sqrt{2}}{2}-e^{-\frac{1}{2}})}$. This cubic inequality is easy to be solved. We can get the solution of this inequality by Cardano’s formula, and we can conclude $M(\delta _{2k},p)<1$ when $0< p< p^{*}(A)$, where

51 $$\begin{aligned} p< p^{*}(A)= \biggl(\sqrt[3]{-\frac{w_{2}}{2}+\sqrt{\frac{w_{2}^{2}}{4}+ \frac {w_{1}^{3}}{27}}}+\sqrt[3]{-\frac{w_{2}}{2}-\sqrt{ \frac{w_{2}^{2}}{4}+\frac {w_{1}^{3}}{27}}} \biggr)^{2}. \end{aligned}$$

Therefore, for a given *k*-sparse matrix $X^{*}\in\mathbb {R}^{n\times r}$ and any $0< p< p^{*}$, we have that

52 $$\begin{aligned} \bigl\Vert X^{*}+H \bigr\Vert _{2,p}^{p} =& \bigl\Vert \bigl(X^{*}+H\bigr)_{S^{*}} \bigr\Vert _{2,p}^{p}+ \Vert H_{S^{*C}} \Vert _{2,p}^{p} \\ \geq& \bigl\Vert X^{*} \bigr\Vert _{2,p}^{p}- \Vert H_{S^{*}} \Vert _{2,p}^{p}+ \Vert H_{S^{*C}} \Vert _{2,p}^{p}> \bigl\Vert X^{*} \bigr\Vert _{2,p}^{p}. \end{aligned}$$

The last inequality is the result from the fact $M(\delta_{2k},p)<1$.

The proof is completed. □

Now, we present one example to demonstrate the validation of our main contribution in this paper.

### Example 2

We consider an underdetermined system $AX=B$, where $A \in\mathbb {R}^{12\times13}$

$$A=\left ( \begin{matrix} 1& 0& \ldots& 0 & 0.3 \\ 0 & 1 & \ldots& 0 & 0.3 \\ 0 & \ddots& \ldots& \vdots& 0.3\\ 0& 0& \ldots& 1& 0.3 \end{matrix} \right )\quad \mbox{and} \quad B=\left ( \begin{matrix} 1 & 1\\ 1 & -1\\ \vdots& \vdots\\ 0& 0 \end{matrix} \right ). $$

It is easy to verify that the unique sparse solution to $l_{2,0}$-minimization is

53 $$\begin{aligned} X^{\ast}=\left ( \begin{matrix} 1 & 1 \\ 1 & -1 \\ \vdots& \vdots\\ 0 & 0 \end{matrix} \right ) \end{aligned}$$

and $N(A)=\{ax:a \in\mathbb {R},x=[1,1,\ldots,1,10/3]^{T}\}$.

So the solution to $AX=B$ can be expressed as the following form:

54 $$\begin{aligned} X=\left ( \begin{matrix} 1+s & 1+t \\ 1+s & -1+t \\ s & t\\ \vdots& \vdots\\ \frac{10}{3}s & \frac{10}{3}t \end{matrix} \right ), \end{aligned}$$

where $s,t \in\mathbb {R}$ such that

$$\begin{aligned} \Vert X \Vert _{2,p}^{p}= \bigl(\sqrt {(1+s)^{2}+(1+t)^{2}} \bigr)^{p}+\bigl(\sqrt {(1+s)^{2}+(-1+t)^{2}} \bigr)^{p}+\frac{40}{3} \bigl(\sqrt{s^{2}+t^{2}} \bigr)^{p}. \end{aligned}$$

**Figure 2:**
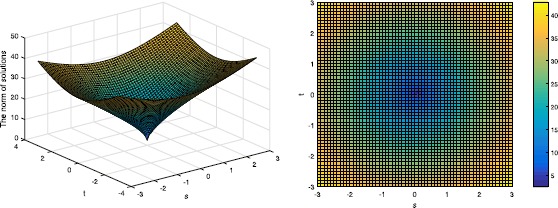
**The norm of the solution in Example** **2** **when p=0.6989, the left one shows the three-dimensional figure and the right one shows the corresponding top view.**

**Figure 3:**
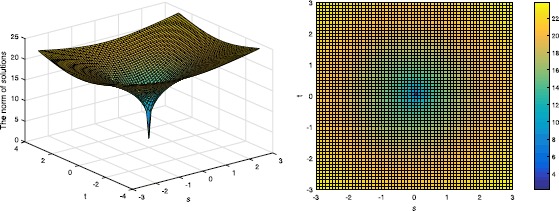
**The norm of the solution in Example** **2** **when** $\pmb{p=0.3}$ **, the left one shows the three-dimensional figure and the right one shows the corresponding top view.**

Then we will verify the result in Theorem 3. It is easy to get that $p^{*}=0.6989$ since $\delta_{4}=0.5612$, and we show the cases when $p=0.6989$ and $p=0.3$ in Figures 2 and 3.

It is obvious that $\Vert X \Vert _{2,p}$ has the minimum point at $s=t=0$, which is the original solution to $l_{2,0}$-minimization.

## Numerical experiment

Although $l_{2,p}$-minimization is difficult to be solved, there are a lot algorithms designed for this problem. In this section, we adopt two excellent algorithms presented in [18] and [21]. The main reason why we choose these algorithms is not only their efficient performance and theoretical results but also the feature that any $p\in (0,1)$ can be applied in these two algorithms. The details of these algorithms are presented in Algorithms 1, 2 and 3. However, we need to underline the choice of the parameters in Algorithms 2 and 3, not the smaller the better, a reasonable *p* has a key role in these algorithms. Therefore, it is urgent for us to see whether these algorithms can do better by using our result.

**Algorithm 1:**
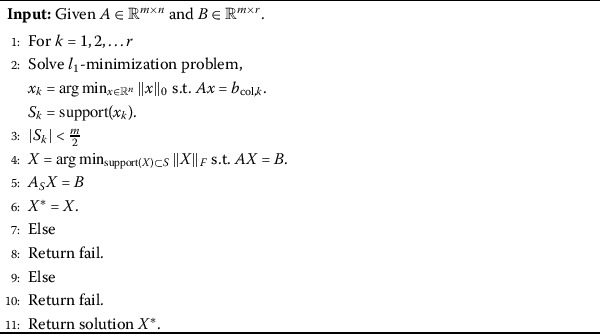
[21]

**Algorithm 2:**
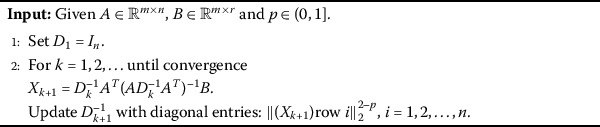
[18]

**Algorithm 3:**
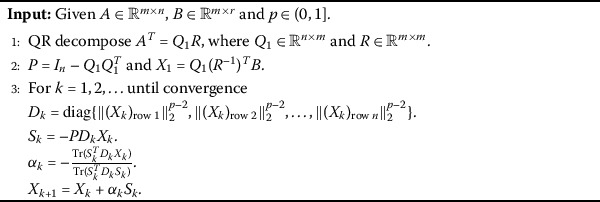
[18]

In order to set reference standards for these $l_{2,p}$-minimization algorithms, we will consider $l_{2,1}$-minimization

55 $$\begin{aligned} \min_{X \in\mathbb{R}^{n\times r}} \Vert X \Vert _{2,1}=\sum _{i=1}^{n} \Vert X_{\text{row } i} \Vert _{2} \quad\text{s.t. }AX=B. \end{aligned}$$

Different to other $p\in(0,1)$, $l_{2,1}$-minimization is a convex optimization problem which can be solved efficiently; especially, this problem can be transformed into a linear programming problem when $r=1$. In this section, we will adopt Algorithm 1 designed for $l_{2,1}$-minimization, and we will adopt a $256\times1024$ measurement *A* with $\delta_{100}=0.8646$, and we can get $p^{*}(A)=0.1507$ by our result.

**Figure 4:**
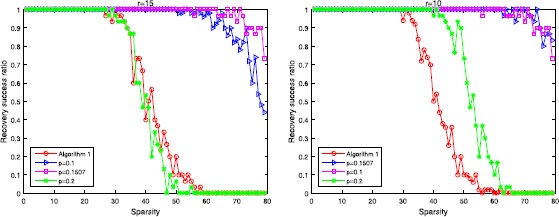
**The result by using Algorithm 2 with different** ***p*** **, the left one is the situation when** $\pmb{r=15}$ **, the right one is the situation when** $\pmb{r=10}$ **.**

**Figure 5:**
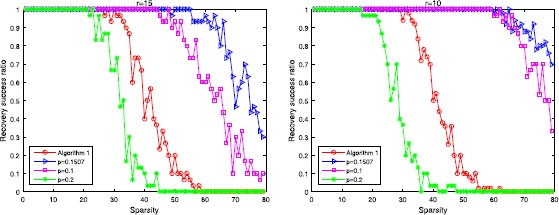
**The result by using Algorithm 3 with different** ***p*** **, the left one is the situation when** $\pmb{r=15}$ **, the right one is the situation when** $\pmb{r=10}$ **.**

Firstly, we look at the relations between sparsity and recovery success ratio. As shown in Figures 4 and 5, the performance of $p=0.1507$ is better than the choice $p=0.2$ and $l_{2,1}$-minimization in both of these two algorithms. The results show us that our result helps these algorithms to increase the efficiency.

Secondly, we look at the relations between the relative error (RE) and sparsity. We define the relative error by

$$\begin{aligned} RE=\frac{ \Vert X^{*}-X_{sol} \Vert _{F}}{ \Vert X^{*} \Vert _{F}}. \end{aligned}$$

**Figure 6:**
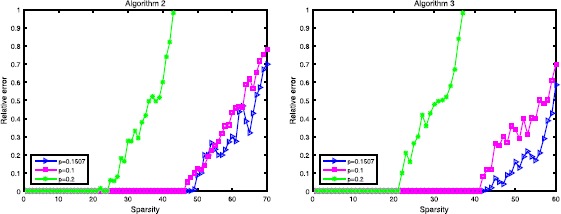
**The performance of Algorithm 2 and Algorithm 3, Algorithm 2 is the left one and Algorithm 3 is the right one.**

As shown in Figure 6, our result performs better than a larger choice. However, we need to emphasize the fact that our result may not be the optimal choice, only a little larger is permitted. In our experiments, the choice of $p=0.16$ is still good, but its performance begins to get worse when $p=0.19$.

## Conclusion

In this paper we have studied the equivalence relationship between $l_{2,0}$-minimization and $l_{2,p}$-minimization, and we have given an analysis expression of such $p^{\ast}$.

Furthermore, it needs to be pointed out that the conclusion in Theorems 2 and 3 is valid in a single measurement vector problem, i.e., $l_{p}$-minimization also can recover the original unique solution to $l_{0}$-minimization when $0< p< p^{\ast}$.

However, the analysis expression of such $p^{\ast}$ in Theorem 3 may not be the optimal result. In this paper, we have considered all the underdetermined matrices $A\in\mathbb{R}^{m \times n}$ and $B\in\mathbb{R}^{m \times r}$ from a theoretical point of view. So the result can be improved with a particular structure of the matrices *A* and *B*. The authors think the answer to this problem will be an important improvement of the application of $l_{2,p}$-minimization. In conclusion, the authors hope that in publishing this paper a brick will be thrown out and be replaced with a gem.
